# Supplementary material for: Nonlinear country-heterogenous impact of the Indian Ocean Dipole on global economies
Source: Nat Commun. 2024 Jun 12;15:5009. doi: 10.1038/s41467-024-48509-5 (PMC11169560; doi:10.1038/s41467-024-48509-5)
Supplement: Supplementary file 1 — Supplementary Information [file 41467_2024_48509_MOESM1_ESM.pdf]

Supplementary Information for

# Nonlinear country-heterogenous impact of the Indian Ocean Dipole on global economies

Wenju Cai, Yi Liu, Xiaopei Lin, Ziguang Li, Ying Zhang, David Newth

*Corresponding authors: Wenju Cai and Ying Zhang

Email: Wenju.Cai@csiro.au and yzhang@ouc.edu.cn

**This PDF file includes:**

Figs. S1 to S13
Tables S1 to S3

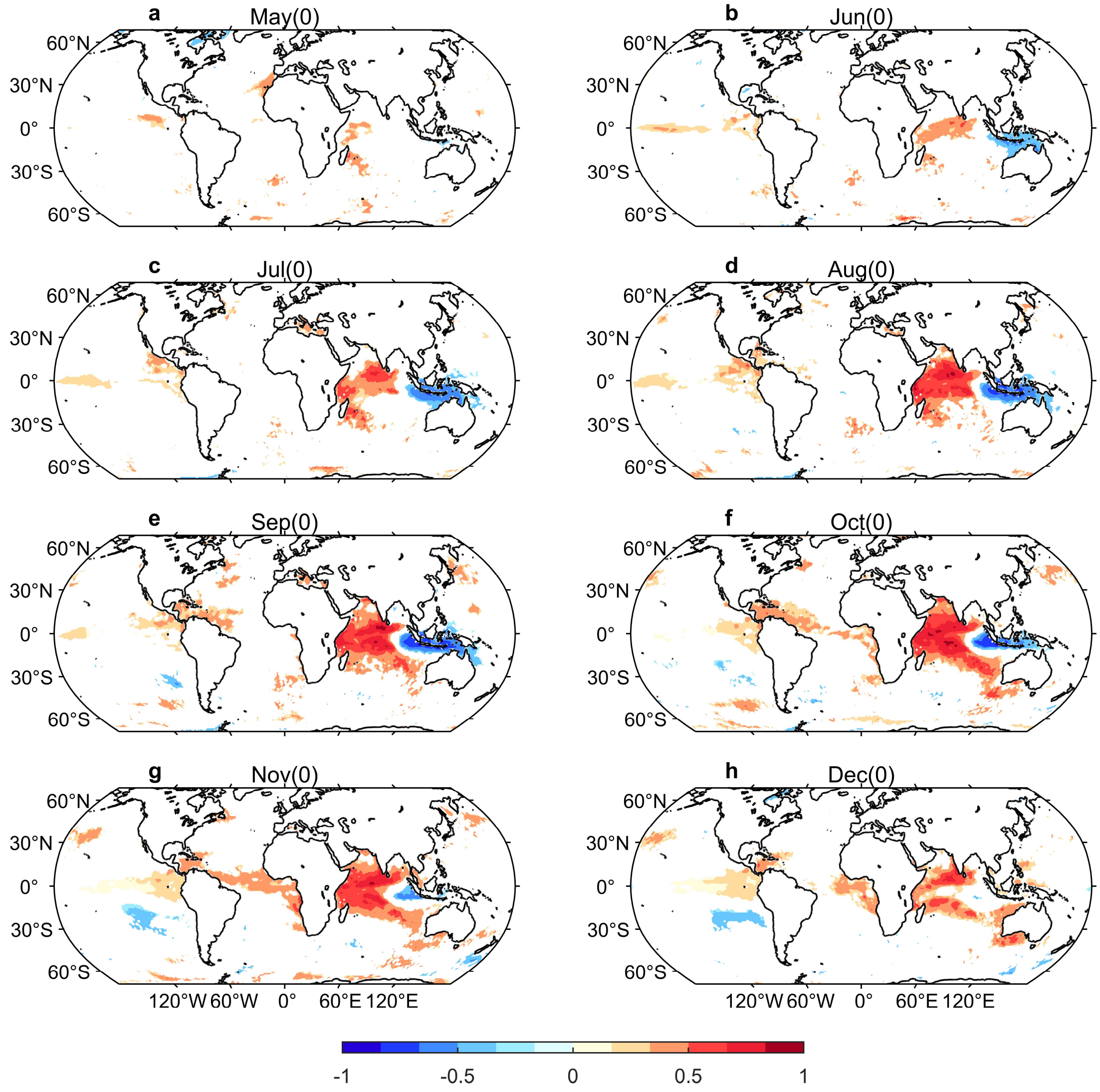


**Fig. S1 | Illustration of removal of ENSO signals in sea surface temperature. a-h** Monthly regression coefficients of sea surface temperature onto the IOD index from May to December in the observations, after removing ENSO signals through a partial regression. Only the regression coefficients above the 95% confidence level are shown. Our partial regression is effective in removing ENSO-related variability. The map is created in the MATLAB computing environment using the M_Map mapping package.

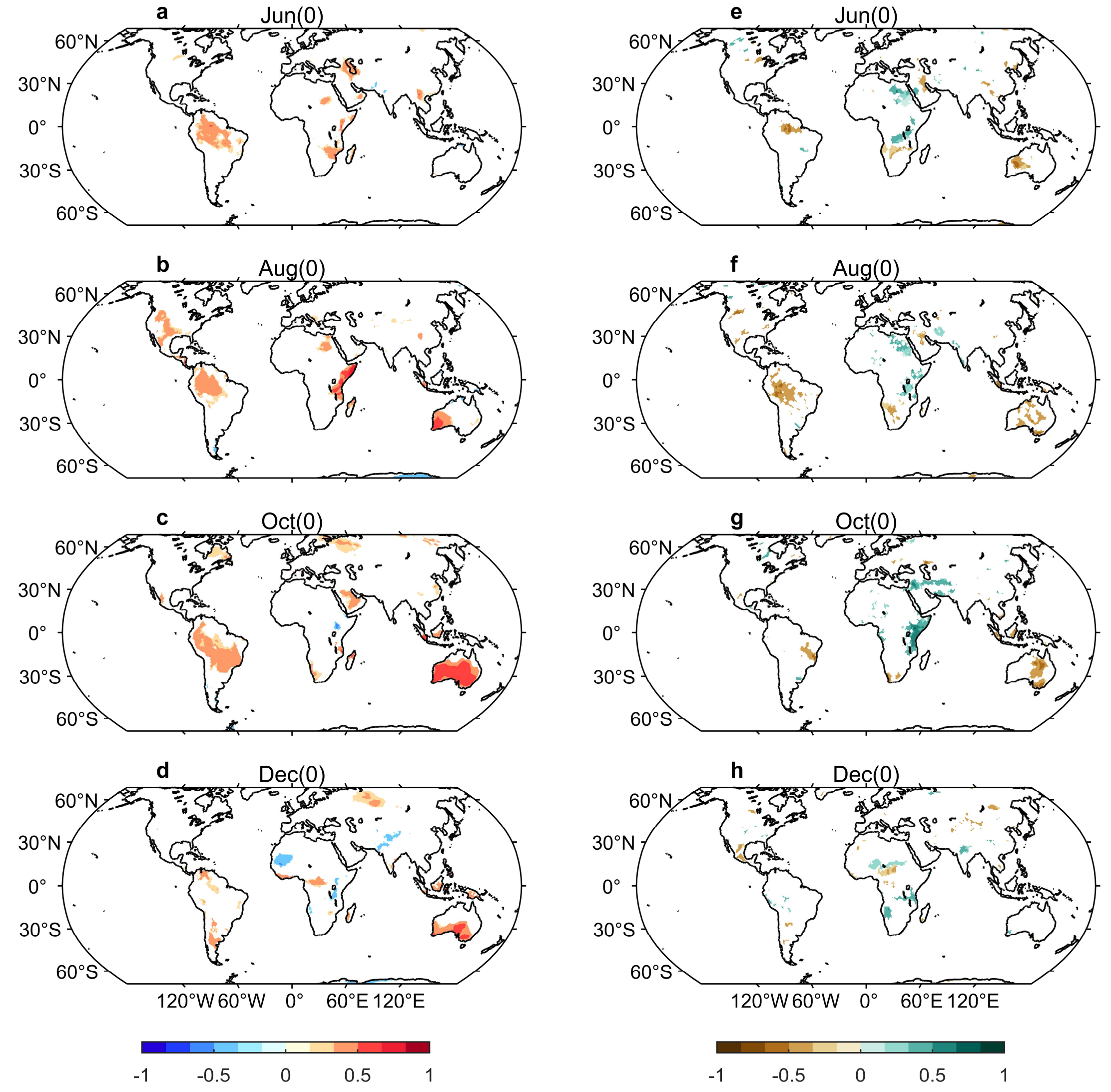


**Fig. S2 | ENSO-free IOD teleconnection of surface air temperature and rainfall. a-d** Monthly regression coefficients of surface air temperature on the IOD index in **a** June, **b** August, **c** October, **d** December in the observations, after removing ENSO signals through a partial regression. Only the regression coefficients above the 95% confidence level are shown. **e-h** Same as **a-d**, but for monthly regression coefficients of rainfall. The IOD affects rainfall and temperature at different times in different regions. The map is created in the MATLAB computing environment using the M_Map mapping package.

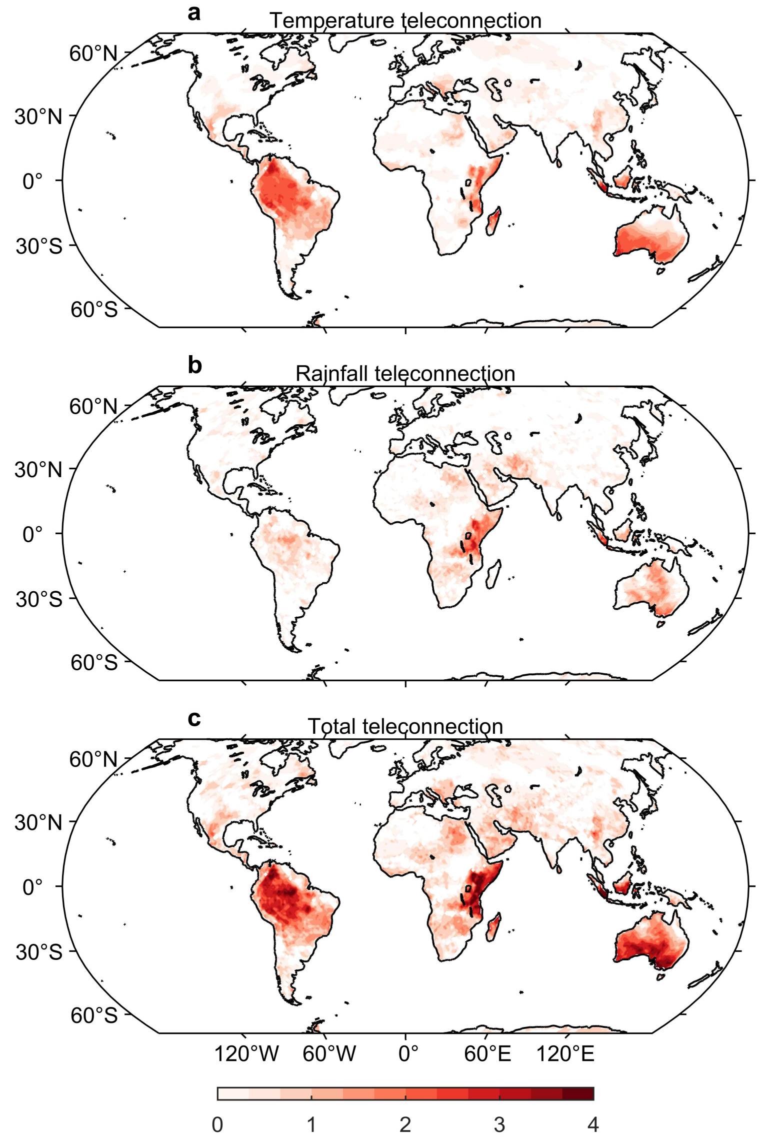


**Fig. S3 | IOD climate teleconnection pattern. a** Surface temperature teleconnection of the IOD, calculated by cumulating the monthly teleconnections of surface air temperature of the IOD (samples shown in Supplementary Fig. S2a-d) and then taking the absolute values. **b** Same as **a**, bur for rainfall teleconnection. **c** Climate teleconnection from theIOD, calculated as the sum of temperature and rainfall teleconnection. Country-specific teleconnections reflect impact from the IOD. The map is created in the MATLAB computing environment using the M_Map mapping package.

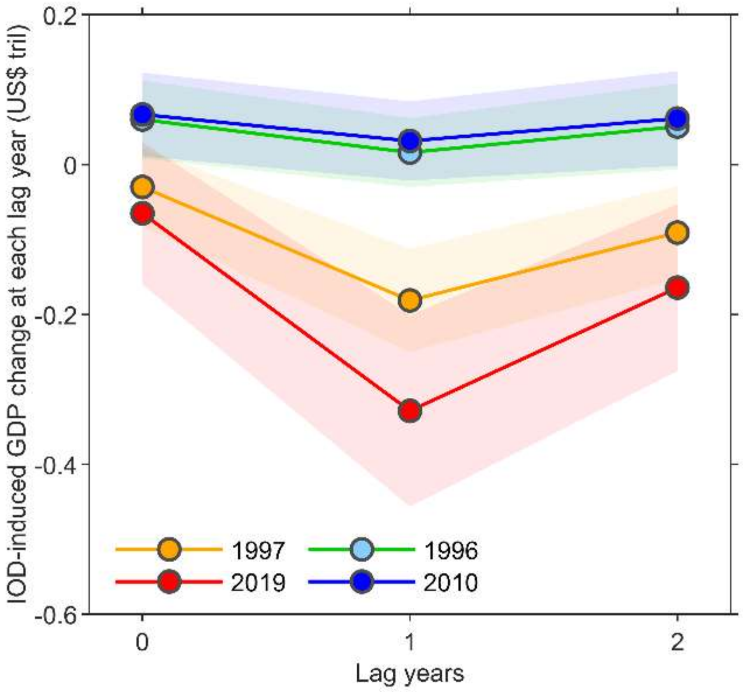


**Fig. S4 | IOD-induced global GDP change at each lag year.** Individual-year effect of two strong pIOD events in 1997 (yellow) and 2019 (red), and two strong nIOD events in 1996 (green) and 2010 (blue) on global GDP cumulating from year 0 (an IOD occurrence year) to year 2. Shadings indicate the 95% confidence interval based on the Bootstrap method.

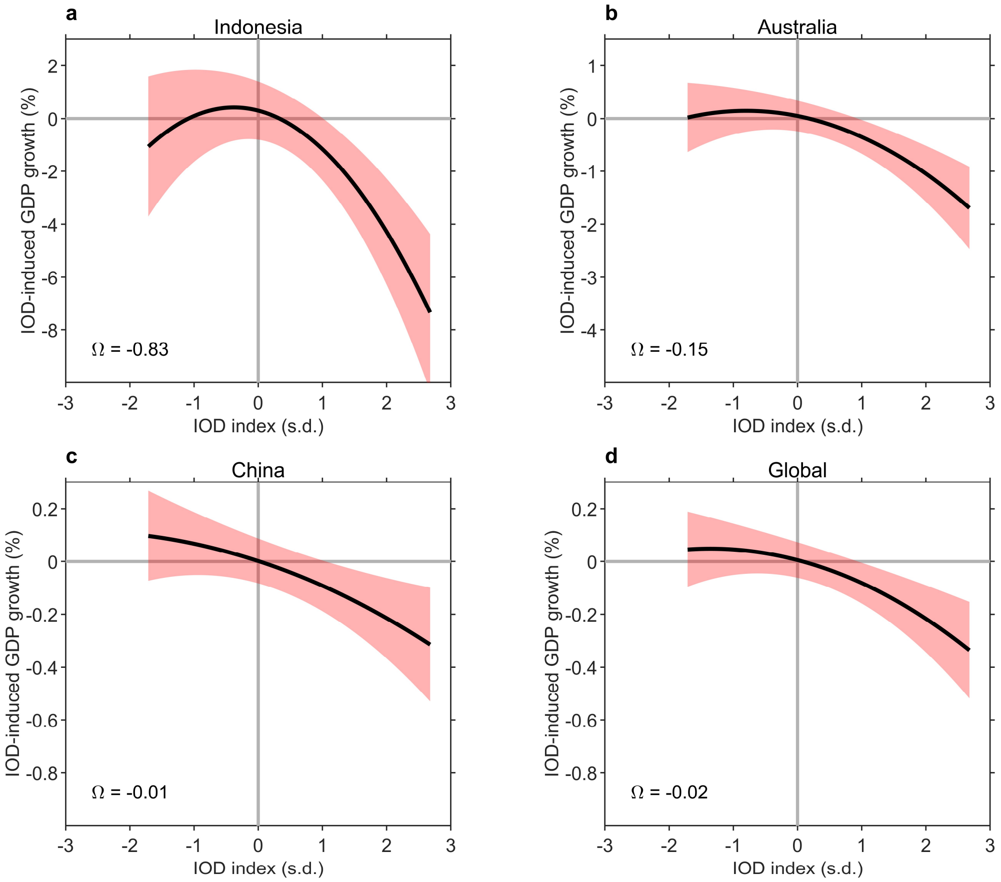


**Fig. S5 | Nonlinear economic impact of the IOD on sample countries and the globe. a-d** Nonlinear relationship between the IOD index time series and time series of its impact on economic growth of **a** Indonesia, **b** Australia, **c** China, **d** the globe. At any year the value of economic impact includes combination of the contemporaneous (year 0) and growth (years 1 and 2) effects; the IOD time series is shifted forward by one year to maximize the coherence, taking into account that the maximum impact occurs at year 1. Shading indicates the 95% confidence interval based on a Bootstrap method (see 'Bootstrap tests' in Methods). The nonlinearity is shown as **Ω**. The impact of the IOD on economies is nonlinear, more so in countries with a greater teleconnection.

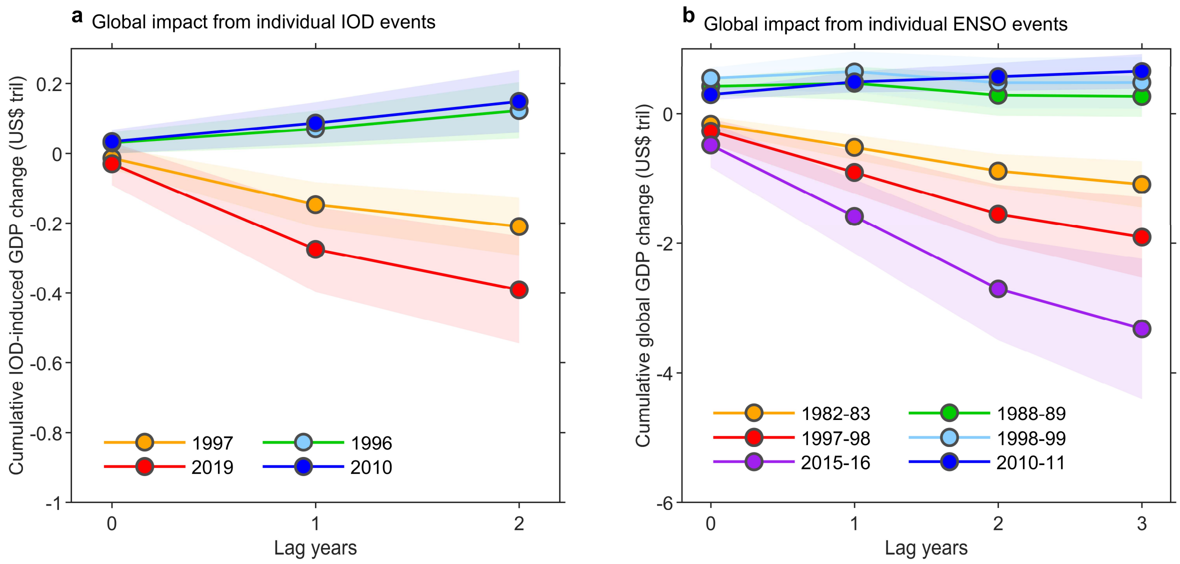


**Fig. S6 | Comparison of economic impacts between observed major ENSO and the IOD events.** **a** Same as Fig. 1c, but estimated by a combined model incorporating both the IOD's country-heterogeneous impact and ENSO's common impact (see 'Empirical econometric model' in Methods). **b** Cumulative effect of three major extreme El Niño events in 1982/83 (yellow), 1997/98 (red) and 2015/16 (purple) on the global GDP cumulating from year 0 (ENSO occurrence year) to year 3, also estimated by the combined model. Shadings indicate the 95% confidence interval based on the Bootstrap method.

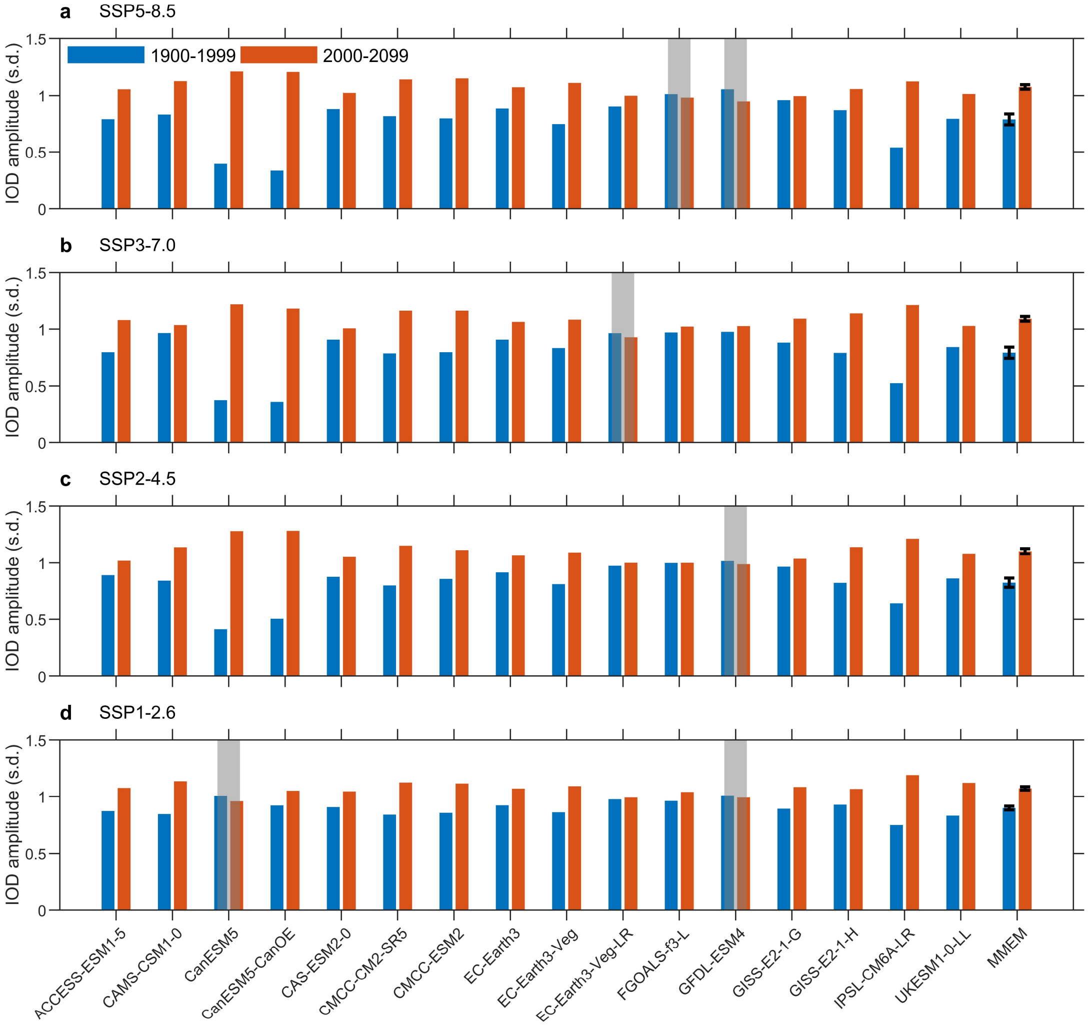


**Fig. S7 | Inter-model consensus on increased IOD amplitude under four IPCC scenarios. a-d** IOD amplitude over the 20th century (1900-1999, blue bars) and 21st century (2000-2099, red bars) for **a** SSP5-8.5, **b** SSP3-7.0, **c** SSP2-4.5, **d** SSP1-2.6 scenarios. Grey shadings indicate models do not project an increase. Error bars for multi-model ensemble refer to the standard deviation of inter-model spread in the 20$^{th}$ and 21$^{st}$ century based on a Bootstrap method. The IOD amplitude is projected to increase under greenhouse warming in plausible emission scenarios.

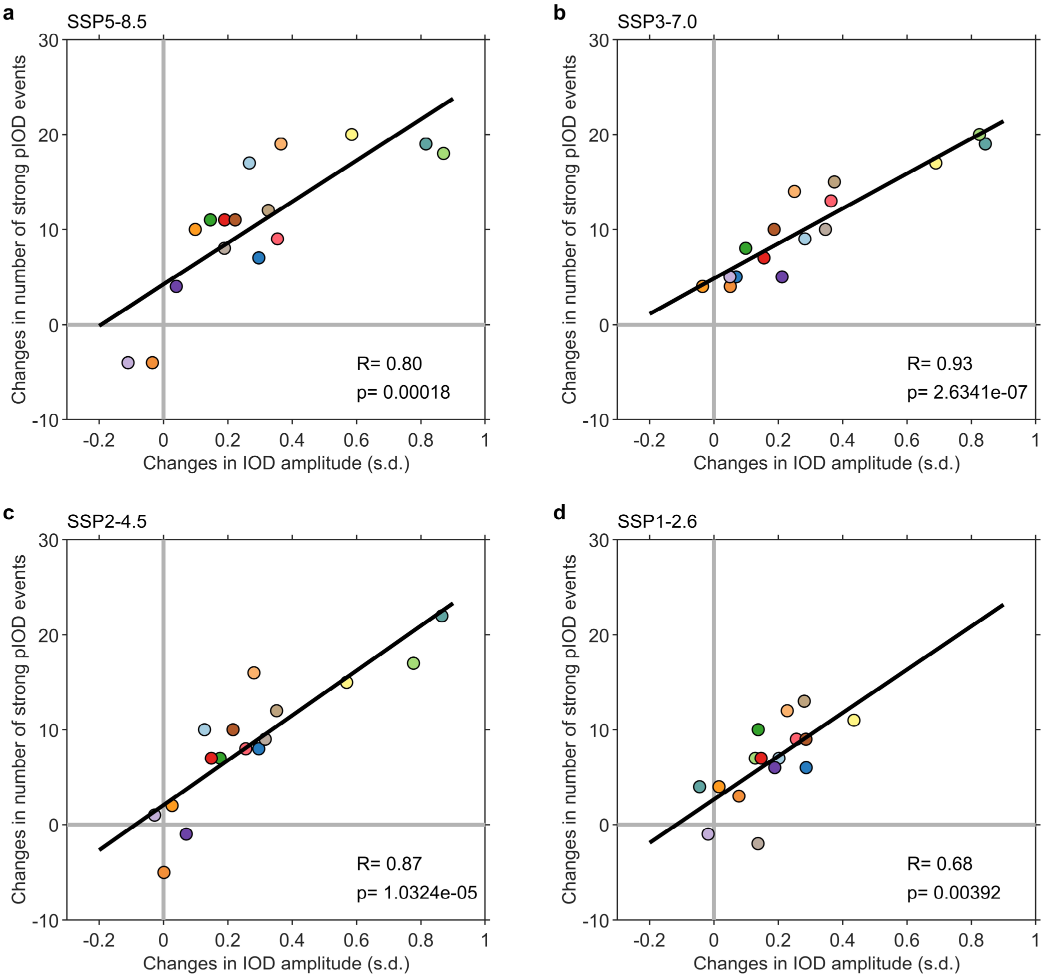


**Fig. S8 | Inter-model relationship between increased IOD amplitude and strong pIOD events under four IPCC scenarios. a-d** Relationship between the changes in IOD amplitude and the changes in the number of strong pIOD events (SON PC1 > 1.5 s.d.) from 20$^{th}$ century to 21$^{st}$ century. Each dot refers to an individual CMIP6 model. A linear fitting is shown as black line for each scenario, together with the correlation coefficient (R) and p-value. The projected increase in IOD amplitude translates to more frequent strong-pIOD events.

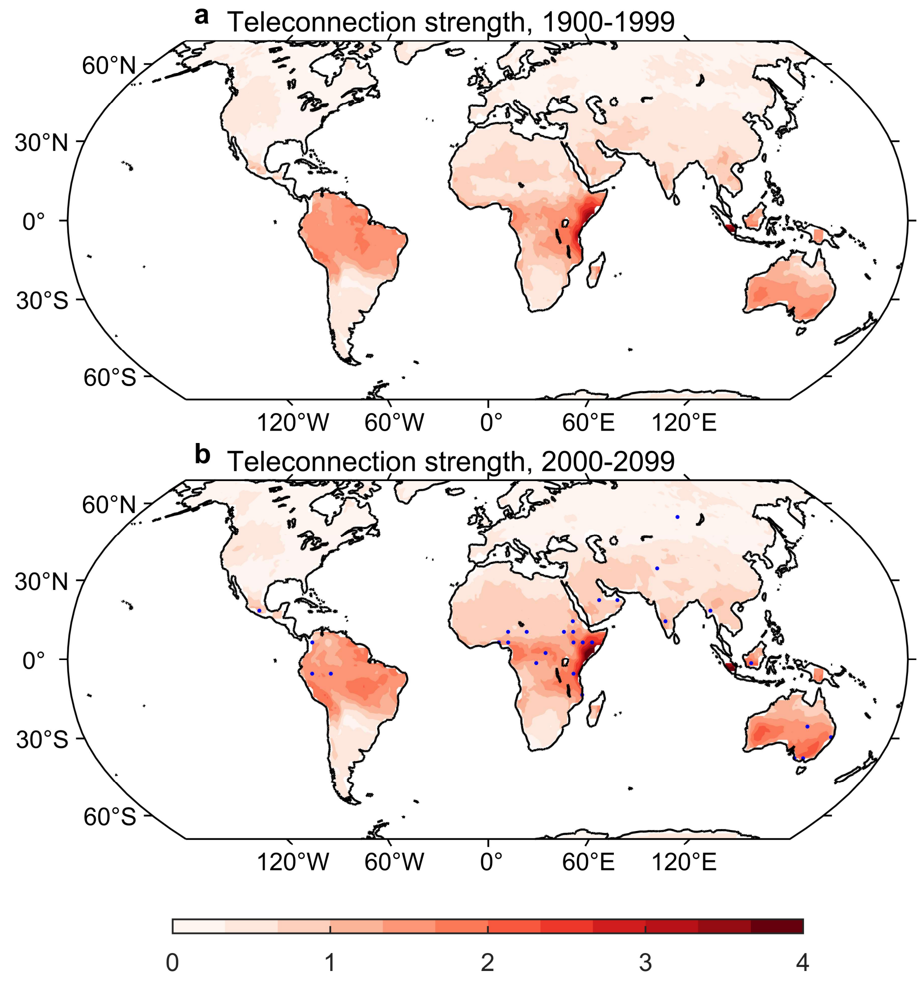


**Fig. S9 | Multi-model ensemble IOD climate teleconnection. a-b** Multi-model ensemble mean of IOD climate teleconnection in **a** the 20th century and **b** the 21st century. Stippled area in **b** indicates where the multi-model ensemble mean change from the 20th century to the 21st century is of inter-model consensus, in which more than 80% models simulate the same sign of changes with the multi-model ensemble mean change. The is little change in the IOD teleconnections under greenhouse warming. The map is created in the MATLAB computing environment using the M_Map mapping package.

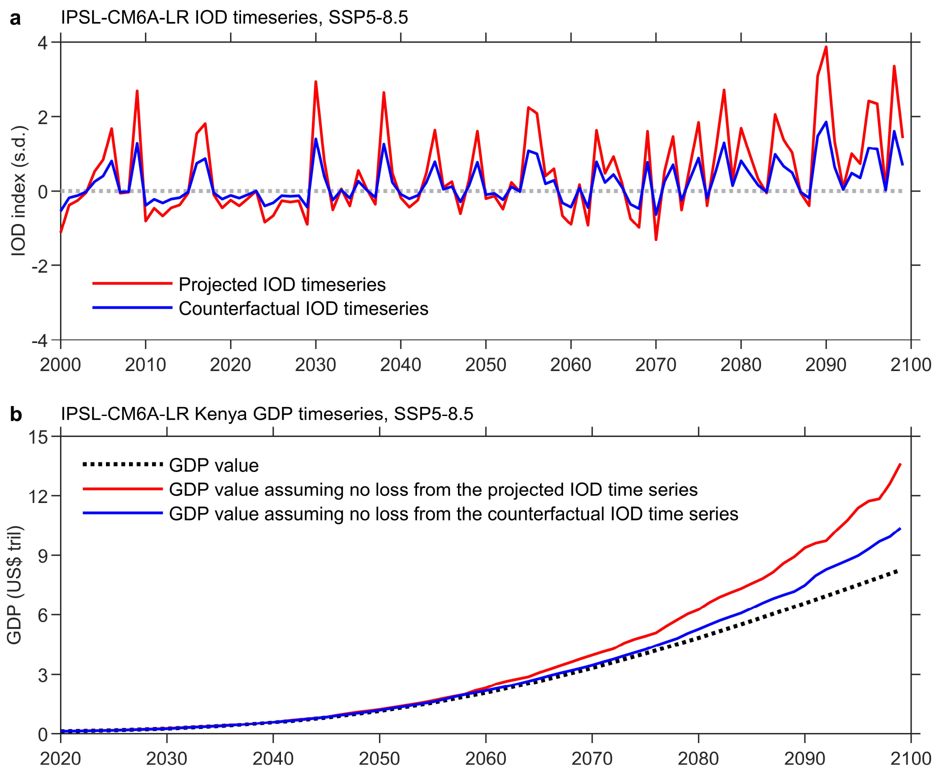


**Fig. S10 | Assessing impact on GDP value from changing IOD. Shown are examples using Kenya, the SSP5-8.5 scenario, and IPSL-CM6A-LR model. a** Timeseries of projected (red) and counterfactual (blue) IOD index for the 21$^{st}$ century under the SSP5-8.5 scenario using IPSL-CM6A-LR as an example. The counterfactual IOD is constructed by scaling the amplitude of the projected 21$^{st}$ to that of the 20$^{th}$ century. **b** Timeseries of Kenya GDP based on time series of the original (black dashed line) and modified growth rates by the projected (red solid line) and the counterfactual (blue solid line) IOD time series. Specifically, we use time series of growth from the projected and the counterfactual IOD time series, each including the contemporaneous and lagged effects, to modify the SSP economic growth time series. The GDP value is calculated by compounding the growth rate from year 2020, at which time the GDP value as taken as the projected. There is a greater loss from the IOD in a future world in which IOD variability increases.

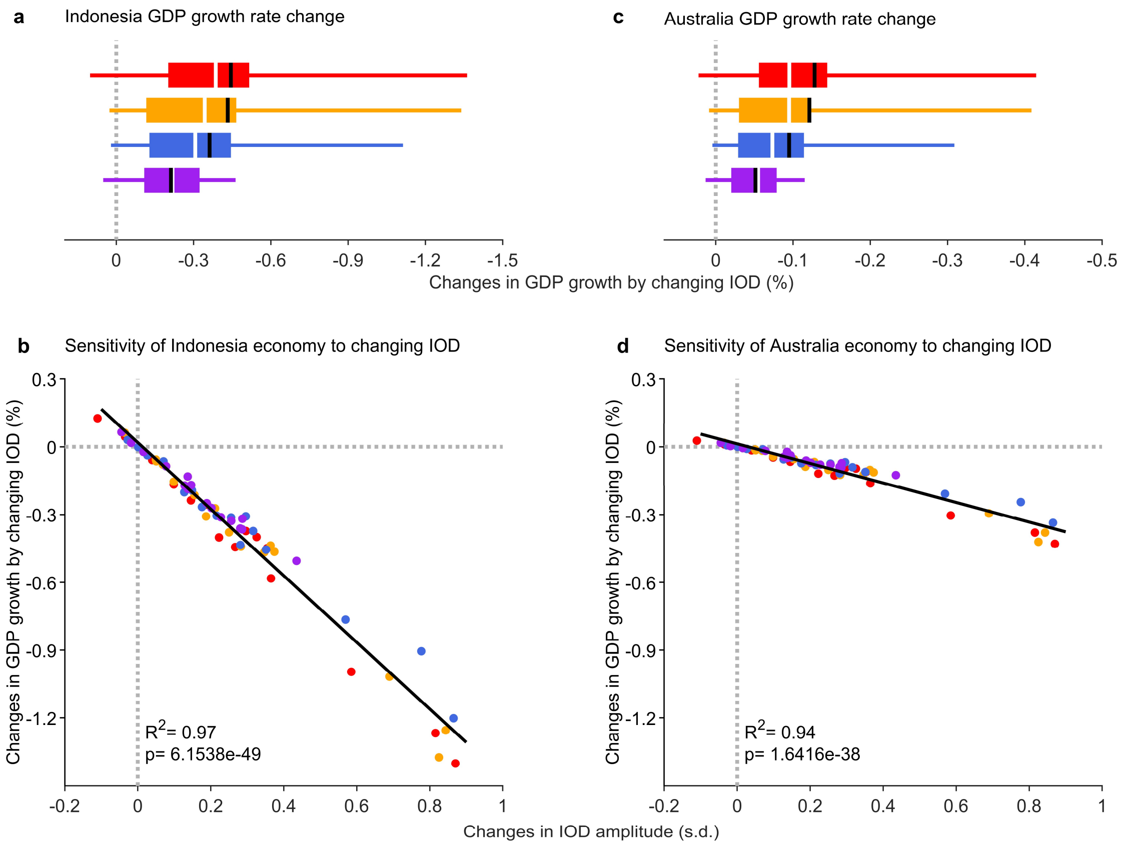


**Fig. S11 | Projected loss to economic growth from changing IOD in Indonesia and Australia.** **a** Changes in century-averaged GDP growth rate from changing IOD for Indonesia, obtained as difference between results derived from the projected and counterfactual IOD time series over the 21$^{st}$ century, with the counterfactual having the same event sequence but the amplitude is scaled to that over the 20$^{th}$ century. **b** Relationship between changes in IOD amplitude (21$^{st}$ century minus 20$^{th}$ century) and changes in Indonesia GDP growth from the changing IOD amplitude. The R square and p-value of a linear fit are given. **c-d** Same as **a-b**, but for Australia.

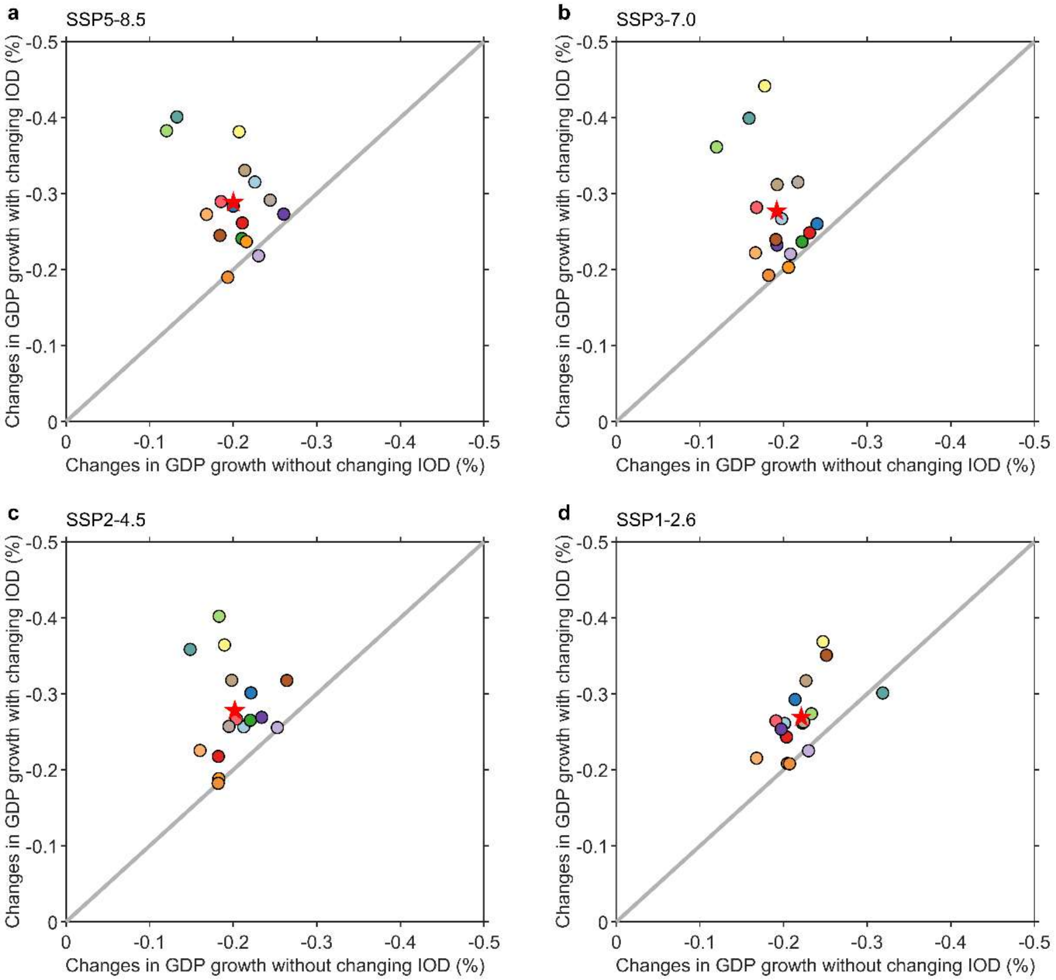


**Fig. S12 | Comparison of IOD-induced global GDP loss with and without IOD changes. a-d**, pIOD-induced loss of global GDP growth with projected changing IOD (y-axis) and counterfactual IOD (x-axis), averaged in the period of 2020-2099 under the **a** SSP5-8.5, **b** SSP3-7.0, **c** SSP2-4.5, and **d** SSP1-2.6 scenario. A pIOD is defined as SON IOD index exceeds 1 s.d. Coloured dots indicate different climate models. Red pentagrams indicate the multi-model ensemble mean. The multimodel average for **a-d** (with, without) IOD changes are (-0.29%, -0.20%), (-0.28%, -0.19%), (-0.28%, -0.20%), and (-0.27%, -0.22%), for the **a** SSP5-8.5, **b** SSP3-7.0, **c** SSP2-4.5, and **d** SSP1-2.6 scenario, with a 45%, 47%, 40%, and 35% increase, respectively.

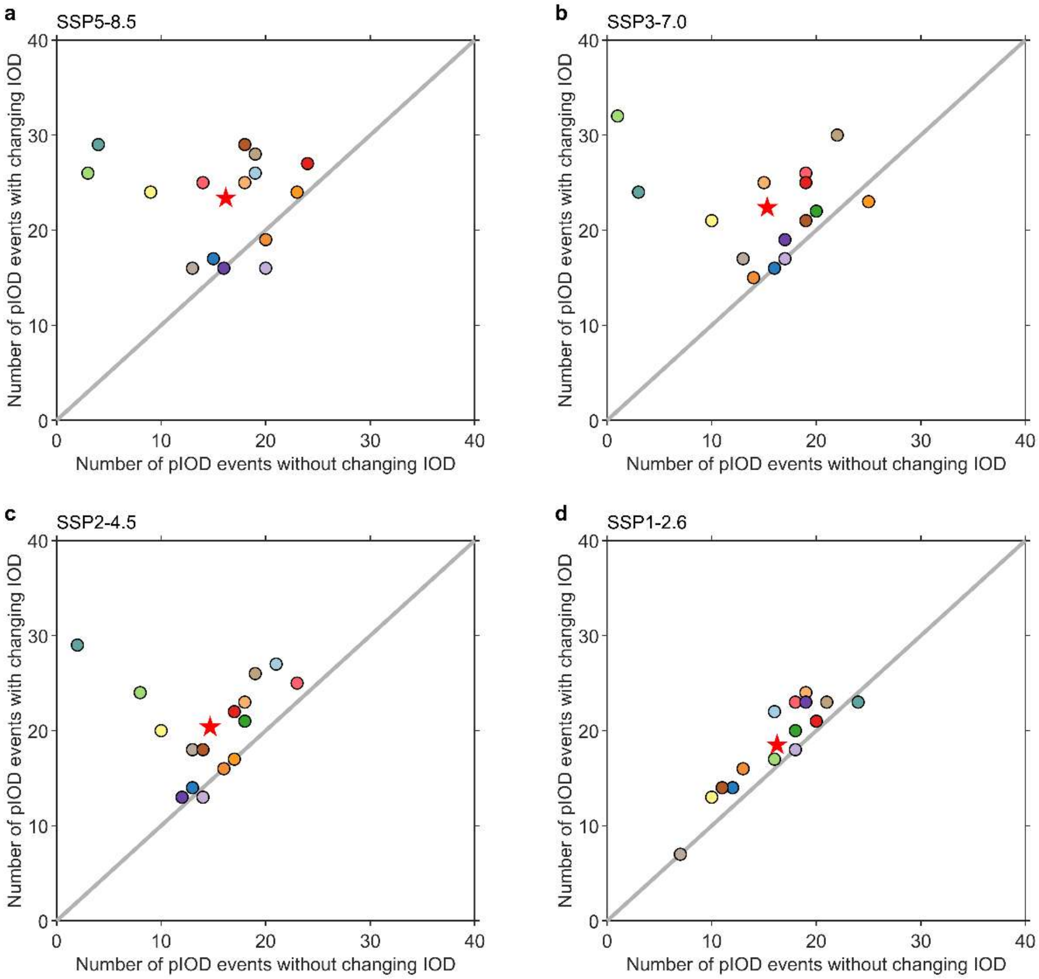


**Fig. S13 | Comparison of numbers of pIOD events with and without IOD changes. a-d**, Same as Fig. S11, but for numbers of pIOD events with projected changing IOD (y-axis) and counterfactual IOD (x-axis), averaged in the period of 2020-2099 under the **a** SSP5-8.5, **b** SSP3-7.0, **c** SSP2-4.5, and **d** SSP1-2.6 scenario. The multimodel average for **a-d** (with, without) IOD changes are (23.4, 16.2), (22.4, 15.3), (20.4, 14.7), and (18.5, 16.3), for the **a** SSP5-8.5, **b** SSP3-7.0, **c** SSP2-4.5, and **d** SSP1-2.6 scenario, with a 44%, 46%, 39%, and 14% increase, respectively.

**Table S1 | Regression coefficients in econometric models with different years of delayed impact.** Values in the brackets denote the standard error of each coefficient. Superscript *, ** and *** indicate the estimate of coefficient is statistically significant above the 90%, 95% and 99% confidence level, respectively. We use the model with three years of lagged effect, but we focus on the effect from year 0 to year 2.

| | **Lag 0** | **Lag 1** | **Lag 2** | **Lag3** |
|---|---|---|---|---|
| $\alpha_{1,0}$ | $-7.12\times10^{-4}$ ($7.92\times10^{-4}$) | -0.0011 ($7.97\times10^{-4}$) | -0.0015* ($8.34\times10^{-4}$) | -0.0015* ($8.51\times10^{-4}$) |
| $\alpha_{1,1}$ | | -0.0013 ($8.45\times10^{-4}$) | -0.0016* ($8.57\times10^{-4}$) | -0.0018* ($9.01\times10^{-4}$) |
| $\alpha_{1,2}$ | | | -0.0015 ($8.82\times10^{-4}$) | -0.0017* ($9.00\times10^{-4}$) |
| $\alpha_{1,3}$ | | | | $-7.15\times10^{-4}$ ($9.03\times10^{-4}$) |
| $\alpha_{2,0}$ | $3.56\times10^{-4}$ ($2.54\times10^{-4}$) | $2.99\times10^{-4}$ ($2.57\times10^{-4}$) | $3.29\times10^{-4}$ ($2.60\times10^{-4}$) | $3.43\times10^{-4}$ ($2.60\times10^{-4}$) |
| $\alpha_{2,1}$ | | $-7.20\times10^{-4}$*** ($2.55\times10^{-4}$) | $-7.63\times10^{-4}$*** ($2.58\times10^{-4}$) | $-7.52\times10^{-4}$*** ($2.62\times10^{-4}$) |
| $\alpha_{2,2}$ | | | $-4.36\times10^{-5}$ ($2.75\times10^{-4}$) | $-4.99\times10^{-5}$ ($2.82\times10^{-4}$) |
| $\alpha_{2,3}$ | | | | $-2.51\times10^{-4}$ ($2.79\times10^{-4}$) |
| $R^2$ (adjusted) | 0.1901 | 0.1938 | 0.1932 | 0.1953 |

**Table S2 | Regression coefficients $\alpha$ from three bootstrap resampling approaches in various lag years in our model that incorporates delayed effect of three years.** Values in the brackets denote s.d. of 1,000 bootstrapped coefficients. Superscript *, ** and *** indicate the estimate of a coefficient is statistically significant above the 90%, 95% and 99% confidence level, respectively.

| | **Sampling by country** | **Sampling by year** | **Sampling by 5-year block** |
|---|---|---|---|
| $\alpha_{1,0}$ | -0.0016* ($5.93\times10^{-4}$) | -0.0015* ($7.24\times10^{-4}$) | -0.0016* ($6.35\times10^{-4}$) |
| $\alpha_{1,1}$ | -0.0018* ($6.69\times10^{-4}$) | -0.0017* ($8.17\times10^{-4}$) | -0.0018* ($8.78\times10^{-4}$) |
| $\alpha_{1,2}$ | -0.0017* ($6.82\times10^{-4}$) | -0.0017* ($7.74\times10^{-4}$) | -0.0017* ($7.65\times10^{-4}$) |
| $\alpha_{1,3}$ | $-7.24\times10^{-4}$ ($7.59\times10^{-4}$) | $-6.94\times10^{-4}$ ($7.90\times10^{-4}$) | $-7.86\times10^{-4}$ ($5.90\times10^{-4}$) |
| $\alpha_{2,0}$ | $4.05\times10^{-4}$ ($2.57\times10^{-4}$) | $3.51\times10^{-4}$ ($1.64\times10^{-4}$) | $3.45\times10^{-4}$ ($1.76\times10^{-4}$) |
| $\alpha_{2,1}$ | $-7.61\times10^{-4}$*** ($1.74\times10^{-4}$) | $-7.49\times10^{-4}$*** ($2.61\times10^{-4}$) | $-7.37\times10^{-4}$*** ($3.09\times10^{-4}$) |
| $\alpha_{2,2}$ | $-2.41\times10^{-5}$ ($2.25\times10^{-4}$) | $-2.42\times10^{-5}$ ($2.12\times10^{-4}$) | $-3.21\times10^{-5}$ ($1.93\times10^{-4}$) |
| $\alpha_{2,3}$ | $-2.50\times10^{-4}$ ($2.28\times10^{-4}$) | $-2.56\times10^{-4}$ ($1.43\times10^{-4}$) | $-2.56\times10^{-4}$ ($1.15\times10^{-4}$) |

**Table S3 | Information of CMIP6 models used in this study.** Names of CMIP6 models, the associated institutions and countries, their ensemble members used in this study (mostly r1i1p1f1, with different ensembles labeled in bold), and IOD skewness under SSP5-8.5. Models in grey are not selected because the IOD skewness is below 33% of the observed skewness.

| CMIP6 Model | Institute, Country | Ensemble used | IOD skewness |
|---|---|---|---|
| ACCESS-CM2 | CSIRO, Australia | r1i1p1f1 | -0.12 |
| ACCESS-ESM1-5 | | r1i1p1f1 | 0.66 |
| AWI-CM-1-1-MR | | r1i1p1f1 | 0.12 |
| BCC-CSM2-MR | BCC, China | r1i1p1f1 | 0.13 |
| CAMS-CSM1-0 | CAMS, China | r1i1p1f1 | 1.34 |
| CanESM5 | CCCMA, Canada | r1i1p1f1 | 1.50 |
| CanESM5-CanOE | | **r1i1p2f1** | 1.74 |
| CAS-ESM2-0 | CAS, China | r1i1p1f1 | 0.99 |
| CESM2-WACCM | NCAR, USA | r1i1p1f1 | -0.06 |
| CMCC-CM2-SR5 | CMCC, Italy | r1i1p1f1 | 1.26 |
| CMCC-ESM2 | | r1i1p1f1 | 1.17 |
| CNRM-CM6-1 | CNRM, France | **r1i1p1f2** | 0.09 |
| CNRM-CM6-1-HR | | **r1i1p1f2** | -0.41 |
| CNRM-ESM2-1 | | **r1i1p1f2** | 0.15 |
| EC-Earth3 | Europe-wide consortium | r1i1p1f1 | 0.77 |
| EC-Earth3-Veg | | r1i1p1f1 | 0.66 |
| EC-Earth3-Veg-LR | | r1i1p1f1 | 0.39 |
| FGOALS-f3-L | CAS, China | r1i1p1f1 | 0.53 |
| FGOALS-g3 | | r1i1p1f1 | -0.25 |
| GFDL-ESM4 | NOAA-GFDL, USA | r1i1p1f1 | 0.78 |
| GISS-E2-1-G | NASA/GISS, USA | **r1i1p1f2** | 0.50 |
| GISS-E2-1-H | | **r1i1p1f2** | 0.81 |
| INM-CM4-8 | INM, Russia | r1i1p1f1 | -0.46 |
| INM-CM5-0 | | r1i1p1f1 | -0.49 |
| IPSL-CM6A-LR | IPSL, France | r1i1p1f1 | 1.58 |
| KACE-1-0-G | NIMS-KMA, Korea | r1i1p1f1 | -0.62 |
| MCM-UA-1-0 | UA, USA | **r1i1p1f2** | -0.08 |
| MIROC6 | JAMSTEC, Japan | r1i1p1f1 | 0.14 |
| MIROC-ES2L | | **r1i1p1f2** | 0.27 |
| MPI-ESM1-2-HR | MPI-M, Germany | r1i1p1f1 | 0.07 |
| MPI-ESM1-2-LR | | r1i1p1f1 | 0.02 |
| MRI-ESM2-0 | MRI, Japan | r1i1p1f1 | -0.25 |
| NorESM2-LM | NCC, Norway | r1i1p1f1 | -0.07 |
| NorESM2-MM | | r1i1p1f1 | 0.11 |
| TaiESM1 | | r1i1p1f1 | 0.04 |
| UKESM1-0-LL | MOHC, UK | **r1i1p1f2** | 0.56 |
